# The effect of youth assertive community treatment: a systematic PRISMA review

**DOI:** 10.1186/s12888-017-1446-4

**Published:** 2017-08-02

**Authors:** Richard Vijverberg, Robert Ferdinand, Aartjan Beekman, Berno van Meijel

**Affiliations:** 1 Department of Child and Adolescent Psychiatry, GGZ-Delfland, PO-box 5016, 2600 GA Delft, The Netherlands; 2 VU Medical Centre/GGZ-InGeest, Amsterdam, The Netherlands; 3 Department of Psychiatry, Amsterdam Public Health Research Institute, VU University Medical Center, Amsterdam, The Netherlands; 4 Inholland University of Applied Sciences, Amsterdam, The Netherlands; 5 Parnassia Psychiatric Institute, The Hague, The Netherlands

**Keywords:** Assertive community treatment, Assertive outreach, Adolescent, Review

## Abstract

**Background:**

During the past decades deinstitutionalisation policies have led to a transition from inpatient towards community mental health care. Many European countries implement Assertive Community Treatment (ACT) as an alternative for inpatient care for “difficult to reach” children and adolescents with severe mental illness. ACT is a well-organized low-threshold treatment modality; patients are actively approached in their own environment, and efforts are undertaken to strengthen the patient’s motivation for treatment. The assumption is that ACT may help to avoid psychiatric hospital admissions, enhance cost-effectiveness, stimulate social participation and support, and reduce stigma.

ACT has been extensively investigated in adults with severe mental illness and various reviews support its effectiveness in this patient group. However, to date there is no review available regarding the effectiveness of youth-ACT. It is unknown whether youth-ACT is as effective as it is in adults. This review aims to assess the effects of youth-ACT on severity of psychiatric symptoms, general functioning, and psychiatric hospital admissions.

**Method:**

A systematic literature search was conducted in PubMed, Cochrane Library, PsychINFO and CINAHL published up to March 2017. To assess methodological quality of the included studies, the Oxford Centre of Evidence-Based Medicine grading system was used.

**Results:**

Thirteen studies were included in this review. There are indications that youth-ACT is effective in reducing severity of psychiatric symptoms, improving general functioning, and reducing duration and frequency of psychiatric hospital admissions.

**Conclusions:**

The current literature on youth-ACT is limited but promising. There are indications that youth-ACT is effective in reducing severity of psychiatric symptoms, improving general functioning, and reducing duration and frequency of psychiatric hospital admissions. The effect of youth-ACT may be comparable with the effect of ACT in adults. Similar as in adult ACT, the studies on youth-ACT found effects that vary from small to large. Randomized experimental research designs are needed to further corroborate effectiveness.

**Electronic supplementary material:**

The online version of this article (doi:10.1186/s12888-017-1446-4) contains supplementary material, which is available to authorized users.

## Background

In many countries, over the past decades, a transition has taken place from inpatient to community mental health care for individuals with a severe mental illness. Assertive Community Treatment (ACT) can be considered the result of this transition [1–3].

ACT [4], the most thoroughly studied type of psychiatric case management in adults [5], is characterized by 9 core elements [6–8]: (a) home-based treatment (obligatory), (b) small caseload (size < 10), (c) patients difficult to reach, (d) transition (from clinic to home) case management, (e) early intervention, (f) psychiatric assessment in the community, (g) family support, (h) reintegration/vocational and educational therapy, (i) pharmacology. ACT teams share responsibility for patients. ACT is characterized by an active team approach which focusses on establishing a solid therapeutic alliance between patients, their relatives, and professionals. Also, efforts are undertaken to strengthen a patient’s motivation for treatment and care [9].

The World Health Organization (WHO) Europe has declared assertive outreach care a necessary alternative for inpatient care. This is because treatment focuses on strengthening the patient’s autonomy by enhancing skills and coping, but also by collaboration with relatives and the broader social network. Even during inpatient treatment, the ACT case manager remains involved, which enhances continuity of care [10]. In Europe, 22 out of 42 countries have policies and/or legislation requiring that individuals with severe mental disorders have access to Assertive Community Treatment or assertive outreach related services [10].

Compared to adults, children and adolescents with severe mental illness are at higher risk of being hospitalized [11–14]. Severe mental illness can be defined as a mental, behavioral, or emotional disorder, that meets the Diagnostic and Statistical Manual of Mental Disorders (DSM) criteria, and which results in serious functional impairment substantially interfering with major life activities [15]. The National Institute for health and Care Excellence (NICE) recommends assertive outreach services for children and adolescents with several severe mental illnesses (see guidelines “Psychosis and schizophrenia in children and young people” [14] and “Bipolar disorder, in adults, children and young people in primary and secondary care” [16]). Horatio, the European Association for Psychiatric Nurses [17], and the Executive Agency for Health and Consumers [18] also recommend ACT services for youths.

Because the implementation of youth-ACT is increasing, it is crucial to evaluate its benefits. ACT has been extensively investigated in adults and various reviews have published positive effects on reducing psychiatric symptoms, improving general functioning and reducing hospitalizations [19–30]. However, to date a systematic review regarding the effectiveness of youth-ACT is not available. It is unknown whether youth-ACT is effective as it is in adults [31].

The aim of the current review is to assess the effects of youth-ACT in three areas: severity of psychiatric symptoms, general functioning, and frequency and duration of psychiatric hospital admissions, since ACT has been primarily developed to positively influence these three outcomes [4].

## Methods

A systematic literature review in compliance with the Preferred Reporting Items for Systematic Reviews and Meta-analyses (PRISMA) guidelines [32] was conducted between August 2016 and March 2017.

### Inclusion criteria

This review included English language papers that focus on patients (a) between 6 to 18 years, (b) who suffer from severe mental illness (mood disorders, behavior disorders, psychotic disorders, and/or substance use disorders), and (c) who are poorly engaged with community mental health services. A treatment program was considered as youth-ACT if it contained at least 6 out of 9 core elements [6–8] and provided information about at least one of the following three possible outcomes of youth-ACT: (a) severity of psychiatric symptoms - defined as the severity of emotional problems, behavior problems, psychotic symptoms, or addiction problems [33]; (b) general functioning - defined in the included manuscripts as general functioning, or level of school attendance, functioning in interpersonal relations and pro-social activities [34]. These constructs are important factors in general functioning and are crucial for the development of the child [35]; (c) psychiatric hospital admission - defined as referral to a psychiatric inpatient health care facility where psychiatric patients reside overnight [36].

### Assessment instruments

Psychiatric symptoms, general functioning, and frequency and duration of psychiatric hospital admissions can be measured from different perspectives [37]. Assessment instruments were classified as follows: clinician-based instruments (clinical judgements by caregivers), client-based instruments (based on opinion of patients or parents), or biometric instruments (measuring biophysical values).

### Literature search

A systematic literature search was conducted in PubMed, Cochrane Library, PsychINFO and CINAHL, in close collaboration with an experienced librarian. In March 2017, the following search string was applied in PubMed:

((Assertive Community Treatment[Title/Abstract] OR Assertive outreach[Title/Abstract] OR (“Community Mental Health Services”[Mesh]) AND (Act OR assertive OR outreach*[Title/Abstract]))) AND (((“Child”[Mesh] OR child*[tiab] OR “Minors”[Mesh] OR “minors”[tiab] OR “Puberty”[Mesh] OR “puberty”[tiab] OR “Pediatrics”[Mesh] OR paediatric*[tiab] OR pediatric*[tiab] OR “Adolescent”[Mesh] OR adolescen*[tiab] OR preschool*[tiab] OR “teenager”[tiab] OR “teenagers”[tiab] OR “teen”[tiab] OR “teens”[tiab] OR youth*[tiab] OR “girlhood”[tiab] OR “girl”[tiab] OR “girls”[tiab] OR “boyhood”[tiab] OR “boy”[tiab] OR “boys”[tiab] OR “school age”[tiab] OR “school-aged”[tiab] OR schoolchild*[tiab] OR “kid”[tiab] OR “kids”[tiab] OR underage*[tiab] OR juvenile*[tiab]))).

The full search strategies of the other databases are available in an Additional file 1.

### Selection procedure

**Fig. 1:**
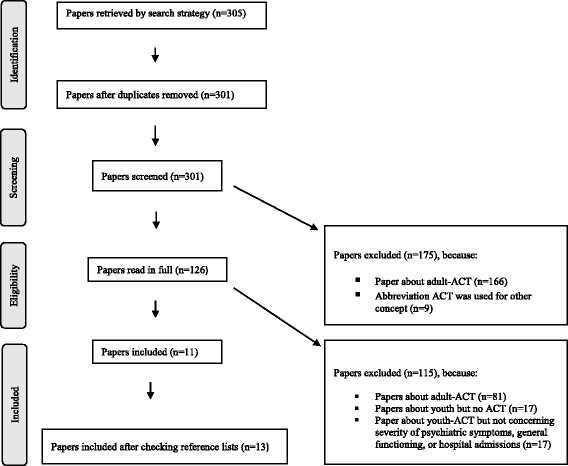
PRISMA Flowchart, inclusion process [32]

Figure 1 shows the selection procedure. English language papers focusing on the effectiveness of youth-ACT, without restrictions concerning research design, were considered for inclusion. After removal of duplicates, papers were independently screened by title and abstract by two authors (RV, RF). To verify papers selected, reference lists of included papers were checked for relevant publications. Disagreements between the reviewers were resolved through discussion. This occurred in 6% of the abstracts. All disagreements related to the decision whether the inclusion criteria were applicable. For example, the abstract did not mention the age category of the included patients. In these cases, the full text of a manuscript was read by RV, after which follow-up discussion took place with RF, until consensus was reached. Papers providing information on the effects of youth-ACT on severity of psychiatric symptoms, general functioning, or frequency and duration of psychiatric hospital admissions were included.

### Data extraction

Data extraction was conducted by the first author (RV), and checked by the second author (RF). Data were extracted using a form containing the following items: author, country of origin, study design, inclusion/exclusion criteria, aim, time-period in which the study was conducted, setting of the study, patient characteristics, sample size, content of the ACT-program, duration or frequency of interventions, assessment instruments, outcomes, and conclusions. As a result, an overview was created that facilitated comparison of study designs and results.

### Quality appraisal

The Oxford Centre of Evidence-Based Medicine grading system was used to assess methodological quality of the individual studies by a standardized approach [38]. The quality of studies was assessed to determine the strength of the scientific evidence of the outcomes of the different studies. The Oxford Centre of Evidence Based Medicine grading system was used because it is a widely adopted systematic hierarchy of the quality of medical research evidence. Quality was classified according to the level of evidence [38]. Studies were classified as follows. High level of evidence: 1a (=systematic review of randomized controlled trials (RCTs)), 1b (=individual RCT), 1c (=all or none RCT). Moderate level of evidence: 2a (=systematic review of cohort studies), 2b (=cohort study or low quality RCT), 2c (=outcome research or ecological studies), 3a (=systematic review of case-control studies), 3b (=case-control study). Low level of evidence: 4 (=case series). Very low level of evidence: 5 (=expert opinion) (Table 1).

**Table 1:** Overview included studies

Reference^a^	Study design (time-frame)	Core elements of youth-ACT		Problems treated	N^b^	Age (years)	Gender (%)	Ethnic group (%)		Team staff	Level of evidence^c^
Adrian & Smith (2014) GBR [46]	Pre-post test (2001–2011)	Home-based treatment:	Yes	Serious mental illness in crisis, admission is considered	287	Range: 12–17 Mean: 16	Boys: 38 Girls: 62	White: Black: Asian: Other:	73 13 4 10	Psychiatrist Nurse practitioner Psychologist Support worker Administrator	2b^c^
		Small caseload (size < 10):	NR^d^								
		Hardly assessable patients:	Yes								
		Transition case management:	Yes								
		Early intervention:	Yes								
		Psychiatric assessment at home:	Yes								
		Family support:	Yes								
		Therapy^e^:	Yes								
		Pharmacology:	Yes								
Ahrens et al. (2007) USA [47]	Pre-post test (1998–2000)	Home-based treatment:	Yes	Long-term mental healthcare needs in transition to adulthood	15	Range: 15–20 Mean: 17	Boys: 80 Girls: 20	White: Black:	80 20	Interdisciplinary, not specified	2b^c^
		Small caseload (size < 10):	NR^d^								
		Hardly assessable patients:	Yes								
		Transition case management:	Yes								
		Early intervention:	Yes								
		Psychiatric assessment at home:	Yes								
		Family support	Yes								
		Therapy^e^:	Yes								
		Pharmacology:	Yes								
Baier et al. (2013) CHE [6]	Pre-post test (2009–2010)	Home-based treatment:	Yes	Psychiatric symptoms and avoiding outpatient care	35	Range: 13–18 Mean: 16	Boys: 43 Girls: 57	NR^d^		Child psychiatrist Social workers Nurses	2b^c^
		Small caseload (size < 10):	Yes								
		Hardly assessable patients:	Yes								
		Transition case management:	Yes								
		Early intervention:	Yes								
		Psychiatric assessment at home	Yes								
		Family support:	Yes								
		Therapy^e^:	Yes								
		Pharmacology:	Yes								
Chai et al. (2012) AUS [48]	Pre-post test (2006–2008)	Home-based treatment:	Yes	Psychiatric symptoms and avoiding outpatient care	59	Range: 11–17 Modal: 15	Boys: 32 Girls: 68	NR^d^		Psychiatrist Social workers “Clinicians”	2b^c^
		Small caseload (size < 10):	Yes								
		Hardly assessable patients:	Yes								
		Transition case management:	Yes								
		Early intervention:	Yes								
		Psychiatric assessment at home:	NR^d^								
		Family support:	Yes								
		Therapy^e^:	Yes								
		Pharmacology:	Yes								
Godley et al. (2002) USA [49]	RCT (1999–2001)	Home-based treatment:	Yes	Alcohol/ drugs dependence or abuse	114	Range: 12–17 Mean: 16	Boys: 80 Girls: 20	White: Black: Other:	74 17 9	Case manager (not specified)	2b^c^
		Small caseload (size < 10):	Yes								
		Hardly assessable patients:	Yes								
		Transition case management:	Yes								
		Early intervention:	Yes								
		Psychiatric assessment at home:	Yes								
		Family support:	Yes								
		Therapy^e^:	Yes								
		Pharmacology:	NR^d^								
Godley et al. (2006) USA [50]	RCT (1999–2003)	Home-based treatment:	Yes	Alcohol/ drugs dependence or abuse	183	Range: 12–18 Mean: 16	Boys: 71 Girls: 29	White: Black: Other:	73 18 9	Case manager (not specified)	2b^c^
		Small caseload (size < 10)	Yes								
		Hardly assessable patients:	Yes								
		Transition case management:	Yes								
		Early intervention:	Yes								
		Psychiatric assessment at home:	Yes								
		Family support:	Yes								
		Therapy^e^:	Yes								
		Pharmacology:	NR^d^								
Godley et al. (2010) USA [51]	RCT (2002–2007)	Home-based treatment:	Yes	Alcohol /drugs dependence or abuse	320	Range: 12–18 Mean: 16	Boys: 76 Girls: 24	White: Black: Other:	73 13 14	Case manager (not specified)	2b^c^
		Small caseload (size < 10):	No								
		Hardly assessable patients:	Yes								
		Transition case management:	Yes								
		Early intervention:	Yes								
		Psychiatric assessment at home:	Yes								
		Family support:	Yes								
		Therapy^e^:	Yes								
		Pharmacology:	NR^d^								
Godley et al. (2015) USA [52]	RCT (2004–2008)	Home-based treatment:	Yes	Alcohol /drugs dependence or abuse	305	Range: 12–18 Mean: 16	Boys: 63 Girls: 37	White: Black: Other:	70 12 18	Case manager (not specified)	2b^c^
		Small caseload (size < 10):	NR^d^								
		Hardly assessable patients:	Yes								
		Transition case management:	Yes								
		Early intervention:	Yes								
		Psychiatric assessment at home:	Yes								
		Family support:	Yes								
		Therapy^e^:	Yes								
		Pharmacology:	NR^d^								
McFarlane et al. (2014) USA [40]	Quasi-experimental (2007–2010)	Home-based treatment:	Yes	Risk or early symptoms of psychosis	337	Range: NR^d^ Mean: 17	Boys: 60 Girls: 40	White: Black: Other:	62 9 19	Psychiatrist Nurse practitioner Nurse Occupational therapist Clinical counsellors	2b^c^
		Small caseload (size < 10):	Yes								
		Hardly assessable patients:	Yes								
		Transition case management:	Yes								
		Early intervention:	Yes								
		Psychiatric assessment at home:	Yes								
		Family support	Yes								
		Therapy^e^:	Yes								
		Pharmacology:	Yes								
McGarvey et al. (2014) USA [41]	Pre-post test (2007–2010)	Home-based treatment:	Yes	Substance use or co-occurring disorder and low income	147	Range: 12–18 Mean: 16	Boys: 60 Girls: 40	White: Black: Other:	62 9 19	Psychiatrist Nurse practitioner Nurse Occupational therapist Clinical counsellors	2b^c^
		Small caseload (size < 10):	NR^d^								
		Hardly assessable patients:	Yes								
		Transition case management:	Yes								
		Early intervention:	Yes								
		Psychiatric assessment at home:	NR^d^								
		Family support:	Yes								
		Therapy^e^:	Yes								
		Pharmacology:	Yes								
Schley et al. (2008) AUS [42]	Pre-post test (2000–2004)	Home-based treatment:	Yes	Psychiatric symptoms, with high-risk of self-harm or harming others, avoiding outpatient care	47	Range: 12–18 Mean: 16	Boys: 77 Girls: 23	*City*		Clinical trainers Therapist (not specified)	2b^c^
								White: Black:	52 48		
		Small caseload (size < 10):	Yes								
								*County*			
								White: Black:	63 37		
		Hardly assessable patients:	Yes								
		Transition case management:	Yes								
		Early intervention:	Yes								
		Psychiatric assessment at home:	Yes								
		Family support:	Yes								
		Therapy^e^:	Yes								
		Pharmacology:	NR^d^								
Urben et al. (2015) CHE [8]	Pre-post test (2010–2013)	Home-based treatment:	Yes	Psychiatric symptoms and avoiding outpatient care	98	Range: NR^d^ Mean: 17	Boys: 53 Girls: 47	NR^d^		Psychiatrist Psychologist Social workers Occupational therapist Psychiatric nurse	2b^c^
		Small caseload (size < 10):	Yes								
		Hardly assessable patients:	Yes								
		Transition case management:	Yes								
		Early intervention:	Yes								
		Psychiatric assessment at home:	Yes								
		Family support:	Yes								
		Therapy^e^:	Yes								
		Pharmacology:	Yes								
Urben et al. (2016) CHE [43]	Pre-post test (NR^d^)	Home-based treatment:	Yes	Psychiatric symptoms and avoiding outpatient care	47	Range: 13–18 Mean: NR^d^	Boys: 61 Girls: 39	NR^d^		Psychiatrist Psychologist Social workers Occupational therapist Psychiatric nurse	2b^c^
		Small caseload (size < 10):	Yes								
		Hardly assessable patients:	Yes								
		Transition case management:	Yes								
		Early intervention:	Yes								
		Psychiatric assessment at home:	Yes								
		Family support:	Yes								
		Therapy^e^:	Yes								
		Pharmacology:	Yes								

^a^ ISO codes of representative countries (International Organization for Standardization) [83]

^b^ N = Sample size

^c^ Classification of methodological quality: 2b = RCT, low quality or cohort study (Oxford Centre for Evidence-Based Medicine) [38]

^d^ NR = Not reported

^e^ Therapy = Reintegration/vocational therapy/educational therapy

### Clinical relevance

Although a study can be classified with a high level of evidence, statistically significant effects can still be small, and thereby in many cases of little clinical relevance [39]. To assess clinical relevance, effect sizes (ES) of significant effects were retrieved from the papers as Cohen’s d. If not reported in a paper, Cohen’s d was calculated by the first author (RV) if data for this calculation were provided in the manuscript [40–43].

Effect sizes were categorized as small (≥ 0.2–0.5); medium (> 0.5–0.8); or large (> 0.8) [44].

### Strength of recommendation

The Oxford Centre of Evidence-Based Medicine grading system [38] was used to obtain an overall measure for the strength of a recommendation [45]. Overall conclusions with a high strength of recommendation are of more importance than those with a lower strength. The strength of a recommendation was considered high (grade A) if all studies with respect to a subject were classified with a level of evidence category 1a, 1b or 1c (categories are explained in section Quality Appraisal). The strength of a recommendation was considered moderate (grade B) if studies were classified as level of evidence category 2a, 2b, 2c, 3a or 3b. The strength of a recommendation was considered low (grade C) if studies were classified in category 4 with respect to level of evidence, and very low (grade D) in case of category 5 studies [38].

## Results

The initial search strategy yielded 305 papers (Fig. 1). One hundred and twenty-six papers were selected based on title and abstract. After careful review, 11 studies met the inclusion criteria. Two additional studies were identified following the checking of reference lists of these 11 studies. In total 13 studies were selected for inclusion.

All selected papers contained at least six of the nine core elements of regular ACT and are presented in Table 1. Conducting a meta-analysis was not possible because of the limited number of studies and the variety of outcome variables. Therefore, the results are presented narratively.

### Study designs and level of evidence

To assess the quality of the 13 studies, study designs are specified in Table 1. Most studies had a pre-post design and lacked a control group [6, 8, 41–43, 46–48]. One study used a quasi-experimental design with a control group, but patients were not randomized [40]. Four studies were RCTs that studied a mono-disciplinary variant of youth-ACT with a limited number of sessions [49–52]. Two studies used (partly) the same patients [49, 50]. Since no systematic reviews on youth-ACT have been published to date, none of the included papers achieved high quality ratings. All studies included in this review were found to be of moderate evidence level Grade B (2b).

### Sample

Sample characteristics of all included studies are presented in Table 1. The included studies examined adolescents up to age 18, with a wide variety of psychiatric problems including substance abuse, psychotic, emotional, and developmental problems. Patients received youth-ACT as the only treatment [6, 8, 40, 42, 43, 46–48] or as an aftercare program [49, 50, 52]. The average ages of included patients ranged from 15 to 17 years. One study included 15 patients, some of whom were 19 or 20 years of age [47]. However, because the majority of the included patients in this study were aged 15, 16 or 17 (mean = 16.8, SD ± 1.4), this paper was retained [47]. None of the reviewed studies included children below age 11. Studies were conducted in the United States [40, 41, 47, 49–52], Switzerland [6, 8, 43], Australia [42, 48], and Great Britain [46]. Most studies investigated a Caucasian sample.

Table 1 shows that girls formed a large majority in three samples [6, 46, 48]. In the other samples, boys formed the majority [8, 40–43, 47, 49–52]. In total, 774 girls and 1217 boys were included.

### Measurements

The severity of psychiatric symptoms was assessed using two clinician-based instruments, the Clinical Global Impression Scale (CGI) [53] and the Health of the Nation Outcome Scales Child and Adolescents Mental Health (HoNOSCA) [54, 55]. Client-based instruments used to measure severity of psychiatric symptoms were the Global Appraisal of Individual Needs (GAIN) [56], Structured Interview for Prodromal Syndromes (SIPS) [57], Timeline Follow Back (TLFB) [58], and the Structured Clinical Interview for DSM-IV Axis I Disorders (SCID-I/CV) [59], Urine drug test or breath-analysing tests were used as biometric instruments [41, 49–52]. General functioning was measured with clinician-based instruments: the GAIN [56], Global Assessment of Functioning (GAF) [60], Children’s Global Assessment Scale (CGAS) [61], and Social and Occupational Functioning Assessment Scale (SOFAS) [46] or with a subscale of HoNOSCA [54, 55]. Hospital admissions were assessed by examining medical files [46–48, 52], or by applying a client-based self-developed structured audit questionnaire [42].

### Effect on severity of psychiatric symptoms

Table 2 shows that 11 studies examined the effect of youth-ACT on the severity of psychiatric symptoms [6, 8, 40–43, 46, 49–52]. Positive effects were reported in ten studies (Table 2). Youth-ACT may have the greatest effect on psychotic symptoms, suicidality, self-harm behavior, and emotional problems. One study reported no additional effect (ES = 0.1) when youth-ACT was added to outpatient mental healthcare [51].

**Table 2:** Effect youth-ACT on severity of psychiatric symptoms

Reference	Main results	Psychiatric disorders in sample (%)		Follow-up (months)	Assessment instruments	Effect size^a^ & 95% CI^b^	
Adrian & Smith (2014) [46]	Youth-ACT with hospital care and without hospital care was associated with reductions in severity of psychiatric symptoms. Larger effect sizes were found for psychotic symptoms, ASD and mood disorders than for self-harm, eating, and neurotic disorders	Mood:	33	P-T^c^	HoNOSCA	Reduction HoNOSCA Sum-scores Both groups: Patients that needed inpatient care during ACT treatment: Patients with only ACT:	1.2 (1.1, 1.4) 1.2 (0.9, 1.5) 1.3 (1.1, 1.5)
		Anxiety:	26				
		Psychotic:	21				
		Self-harm:	12				
		ASD:	2				
		Eating:	2				
		Other:	10				
Baier et al. (2013) [6]	Youth-ACT is associated with reduction of psychiatric symptoms	Psychotic:	51	P-T^c^	HoNOSCA	Reduction HoNOSCA Sum-scores: Disruptive behaviour: Hyperactivity: Self-injury: Substance abuse: Hallucinations: Non-organic: Emotional:	1.3 (0.8, 1.8) 0.1 (−0.4, 0.5) 0.3 (−0,1, 0.8) 0.7 (0.2, 1.1) 0.2 (−0.2, 0.6) 1.0 (0.5, 1.4) 0.2 (−0.3, 0.7) 0.8 (0.3, 1.3)
		Schizophrenia:	23				
		Mood:	14				
		Anxiety:	9				
		Conduct:	26				
Godley et al. (2002) [49]	Preliminary outcomes of Godley et al. (2006) [50]. Significantly more abstinent from marijuana in youth-ACT + Usual Continuing Care (UCC) group compared to only UCC	Substance:	100	3	GAIN TLFB Urine tests Breath-analyser Interviews	Alcohol use: Abstinence at follow-up: Marijuana, 3 months:	0.1 (−0.2, 0.4) 0.4 (0.1, 0.8)
Godley et al. (2006) [50]	Significantly more long-term abstinent from marijuana in youth-ACT + Usual Continuing Care (UCC) compared to only UCC	Substance:	100	3, 6, 9	GAIN TLFB Urine tests Breath-analyser Interviews	Abstinence at follow-up: Alcohol, 3 and 9 months: Marijuana, 3 and 9 months both: Other drugs, 3 months: 9 months:	0.1 (−0.2, 0.4) 0.3 (0.0, 0.6) 0.2 (−0.1, 0.5) 0.1 (−0.1, 0.3)
		Mood:	38				
		Anxiety:	38				
		PTSD:	36				
		ADHD:	57				
		Conduct:	67				
Godley et al. (2010) [51]	Youth-ACT had no additional effect on substance disorders compared to outpatient treatment only	Alcohol:	49	3, 6, 9, 12	GAIN substance problem scale Urine tests	Additional effect of youth-ACT in symptom reducing	0.1 (−0.2, 0.4)
		Marijuana:	75				
		Mood:	28				
		Anxiety:	8				
		PTSD:	19				
		ADHD:	34				
		Conduct:	42				
Godley et al. (2015) [52]	Significantly more long-term abstinent from marijuana and alcohol in youth-ACT compared to only Usual Continuing Care (UCC)	Alcohol:	58	3, 6, 9, 12	GAIN substance problem scale Urine tests Breathalyzer	Abstinence at follow-up: Alcohol: 12 months: Marijuana: 12 months: Other drug: 12 months:	0.3 (0.1, 0.8) 0.3 (0.0, 0.6) 0.3 (0.0, 0.6)
		Marijuana:	91				
		Mood:	32				
		Anxiety:	46				
		PTSD:	33				
		ADHD:	49				
		Conduct:	65				
McFarlane et al. (2014) [40]	Youth-ACT was superior in reducing positive, negative, disorganized symptoms and general symptoms in adolescents compared to community care	Substance:	8	6, 12, 24	SIPS SCID-I/CV	Symptom reduction: Positive symptoms: Negative symptoms: Disorganized:	0.6 (0.4, 0.9) 0.3 (0.0, 0.5) 0.4 (0.2, 0.7)
		Mood:	42				
		Anxiety:	8				
		PTSD:	8				
		OCD:	7				
		Psychosis:	13				
McGarvey et al. (2014) [41]	Youth-ACT reduces marijuana use but does not reduce alcohol use	Substance or co-occurring disorder:	NR^d^	3, 6, 12	GAIN Drug tests	Reduction in days marijuana use at follow-up: Boys at 3 months: 6 months: 12 months: Girls at 3 months: 6 month: 12 months: Alcohol use: Boys at 3 months: 12 months: Girls at 3 months: 12 months:	0.6 (0.3, 0.9) 0.7 (0.5, 1.0) 0.6 (0.3, 0.8) 0.4 (0.1, 0.8) 0.7 (0.0, 1.1) 0.6 (0.1, 1.1) 0.2 (0.0, 0.5) 0.2 (0.0, 0.5) 0.1 (−0.4, 0.7) 0.1 (−0.4, 0.6)
Schley et al. (2008) [42]	Pre-treatment compared to post-treatment showed significant reduction in suicidality and deliberate self-harm behaviour	Substance:	31	P-T^c^	Structured audit questionnaire developed by youth-ACT team	Suicidality: Deliberate self-harm:	2.1 (1.4, 2.8) 2.5 (1.7, 3.3)
		Mood:	40				
		Anxiety	22				
		Psychotic:	9				
		ADHD/Disrupt.:	38				
		Eating:	9				
		Other:	18				
Urben et al. (2015) [8]	Reduction in severity of psychiatric symptoms (pre-treatment compared to post-treatment)	Internalizing:	36	P-T^c^	HoNOSCA	Reduction in HoNOSCA-scores: Externalizing scale: Emotional scale	0.3 (−0.1, 0.5) 0.6 (−0.3, 0.8)
		Externalizing:	27				
		Mix:	37				
Urben et al. (2016) [43]	Reduction in severity of psychiatric Symptoms.	Mood:	30	3, 6, 9	HoNOSCA	Reduction in HoNOSCA Sum-scores: Emotional scale:	0.6 (0.0, 1.2) 0.6 (0.0, 1.2)
		Anxiety:	19				
		Conduct disorder:	17				
		Psychosis:	11				
		Personality disorder:	4				

^a^ Effect sizes were computed as Cohen’s d rounded to the first decimal place. Positive effect sizes represents improvement. Small (≥ 0.2–0.5); medium (> 0.5–0.8); large (> 0.8) [44]

^b^ CI = Confidence interval

^c^ P-T = Pre-Post measurement was conducted

^d^ NR = Not reported

#### Psychiatric symptoms in general

Two studies reported large effect sizes of 1.2 and 1.3 respectively [6, 46] and one study a medium effect size (ES = 0.6 [43]) with respect to a decrease of HoNOSCA sum-scores.

#### Emotional problems

Four studies (Table 2) examined the effect of youth-ACT on emotional symptoms [6, 8, 42, 43]. All studies found a significant reduction of emotional problems. In two studies a medium effect size of 0.6 was found, assessed with the HoNOSCA [8, 43]. In the third study a small decrease was found in scores on the HoNOSCA-item non-organic somatic symptoms (ES = 0.2), a medium decrease in self-injuries (ES = 0.7), and a large decrease in mood symptoms (ES = 0.8) and emotional symptoms (ES = 0.8 [6]). The fourth study reported a large effect size in suicidality (ES = 2.1) and deliberate self-harm behavior (ES = 2.5 [42]).

#### Behavioral problems

One study found a small effect (ES = 0.3) for the decrease in externalizing behavior assessed with the HoNOSCA [8]. Another study reported a similar small effect size for the HoNOSCA-item hyperactivity/focus problems (ES = 0.3), but no significant effect size on disruptive and aggressive behaviors (ES = 0.1 [6]).

#### Psychotic problems

Youth-ACT for patients with psychotic disorders was examined in two studies [6, 40]. One study reported small effects with respect to negative symptoms (ES = 0.3) and disorganized symptoms (ES = 0.4) [40] assessed with the SIPS [57]. A medium effect size was reported for positive symptoms (ES = 0.6) using the same instrument [40]. The second study reported a large effect size (ES = 1.0) for the decrease in HoNOSCA item scores regarding hallucinations and delusions [6].

#### Addiction problems

Five studies reported on the effect of youth-ACT on addiction problems, using subscales of the GAIN [6, 41, 49, 50, 52]. Three studies did not find significant reduction in alcohol abuse (ES = 0.1 [41, 49, 50]). Two studies found a small effect size for alcohol abuse [41, 52], however in one study this was found only for boys and not for girls [41]. Four studies found a reduction in cannabis use [41, 49, 50, 52]. Three of these studies, reported small effect sizes for abstinence of cannabis at the 1 month (ES = 0.3) and the 9 month (ES = 0.3) follow-up [49, 50, 52] although two studies used (partly) the same patients [49, 50]. Also, at the 3-month follow-up, a small effect size (ES = 0.2) was found for diminishing use of drugs other than cannabis in one study [50]. No significant effect (ES = 0.1) was found at the 9 month follow-up [50]. One study reported a reduction in days of cannabis use at 3 month follow-up, with a medium effect size for boys (ES = 0.6), and a small effect size for girls (ES = 0.4 [41]). This study also reported a medium effect size for reduction in days of cannabis use for boys and girls at the 6 month follow-up (ES = 0.7 and 0.6 respectively), and the 12 month follow-up (ES = 0.6) for both boys and girls [41].

### Effect on general functioning

Table 3 shows eight studies with information about the effect of youth-ACT on general functioning [6, 40–43, 46, 48, 52]. All studies reported significant improvements. Effect sizes ranged from small to large. Youth-ACT had the largest effect on school attendance and family relations.

**Table 3:** Effect youth-ACT on general functioning

Reference	Main results	Follow-up (months)	Assessment instruments	Effect size^a^ & 95% CI^b^	
Adrian & Smith (2014) [46]	Compared to baseline 50% of the adolescents treated with youth-ACT showed improvement in general functioning according to CGAS score at discharge. Adolescents with psychotic and mood disorders improved more that patients with neurotic disorders	P-T^c^	CGAS	Baseline compared with discharge CGAS-scores: ACT combined with inpatient care: Only ACT:	1.3 (1.0, 1.6) 1.5 (1.3, 1.7)
Baier et al. (2013) [6]	Youth-ACT associated with significant improvement in social functioning measured with HoNOSCA (school attendance, and peer and family relations)	P-T^c^	HoNOSCA	HoNOSCA-scores: Sum-score: Peer relations: Family relations: School attendance:	1.3 (0.8, 1,8) 0.4 (0.0, 0.9) 0.5 (0.0, 1.0) 0.6 (0.1, 1.1)
Chai et al. (2012) [48]	Significant improvement in clinician-rated levels of social functioning. Adolescents treated with youth-ACT showed increase in school attendance	P-T^c^	CGAS School attendance registration form	School attendance:	0.7 (0.4, 1.1)
Godley et al. (2015) [52]	Small significant improvement in pro-social activities. No significant differences in school attendance and family problems	3, 6, 9, 12	GAIN	Pro-social activities:	0.2 (−0.2, 0.4)
McFarlane et al. (2014) [40]	Adolescents with psychotic symptoms treated with youth-ACT showed significantly higher GAF-outcomes, increased school attendance or work (21%) compared to those who received Community Care (7.0%)	6, 12, 24	GAF	GAF-score:	0.3 (0.0, 0.5)
McGarvey et al. (2014) [41]	Decrease in average number of days missing school (5.3 to 2.6 days) or being expelled from school (0.2 to 0.01 days) compared to baseline	3, 6, 12	GAIN	School attendance: Decrease in days expelled from school:	0.7 (0.4, 1.1) 0.6 (0.3, 0.9)
Schley et al. (2008) [42]	Youth-ACT decreased the frequency of violence and crime	P-T^c^	Structured self-developed questionnaire	Crime: Violence:	0.6 (0.1, 1.2) 0.9 (0.3, 1.5)
Urben et al. (2016) [43]	Adolescents treated with youth-ACT showed significant improvements in HoNOSCA social-score which include the items family relations, peer relations and school attendance.	3, 6, 9	HoNOSCA	HoNOSCA Sum score: Social-score: School attendance:	0.6 (0.0, 1.2) 0.8 (0.1, 1.2) 0.8 (0.2, 1.4)

^a^Effect sizes were computed as Cohen’s d rounded to the first decimal place. Positive effect sizes represents improvement. Small (≥ 0.2–0.5); medium (> 0.5–0.8); large (> 0.8) [44]

^b^CI = Confidence interval

^c^P-T = Pre-Post measurement was conducted

Five studies investigated effects on general functioning [6, 40, 43, 46, 52]: One study reported an increase in GAF-score (ES = 0.3 [40]). A second study reported a large increase in CGAS-score (ES = 1.5), with individuals with psychotic, mood, or autism spectrum disorders improving more than those with neurotic disorders, deliberate self-harm, or eating disorders [46]. A third study found a small effect on pro-social activities (ES = 0.2 [52]). A fourth and fifth study reported large (ES = 1.3) and medium (ES = 0.6) effect sizes respectively, with respect to a decrease of HoNOSCA sum-scores [6, 43].

#### School attendance

Six studies examined school attendance [6, 40, 41, 43, 48, 52]. All studies found a significant effect of youth-ACT. Medium effect sizes (ES = 0.6 [6]), (ES = 0.7 [41]), (ES = 0.7 [48]), and (ES = 0.8 [43]) were reported on the HoNOSCA item school attendance, and decrease of average number of days expelled from school (ES = 0.6 [41]). One study found a decrease of part-time school attendance, and non-attendance [40]. One study reported no significant effect on school attendance [52]. For these two studies ES could not be calculated because required data were not reported [40, 52].

#### Interpersonal relations

Two studies examined the effect of youth-ACT on interpersonal relations [6, 52]. One study used subscales of HoNOSCA [6]. Small effect sizes were found for peer relations (ES = 0.4) and family relations (ES = 0.5). The second study reported no significant effect on experienced family problems [52].

### Effect on psychiatric hospital admissions

All five studies reporting the effect of youth-ACT on frequency and duration of psychiatric hospital admissions found a significant effect (Table 4) [42, 46–48, 52].

**Table 4:** Effect youth-ACT on psychiatric hospital admissions

Reference	Main results	Follow-up (months)	Assessment instruments	Effect size^a^ & 95% CI^b^	
Adrian & Smith (2014) [46]	Youth-ACT associated with reduction in length of hospital admission	12	Medical files	NR^c^	
Ahrens et al. (2007) [47]	Reduction in number of hospitalized days. Decrease in total number of days of inpatient psychiatric treatment, forensic treatment or incarceration	24	Medical files	Reduction admission days: Reduction in time in institutions, inpatient psychiatric treatment, and forensic treatment or incarceration:	0.5 (−0.2, 1.3) 0.6 (−0.3, 1.4)
Chai et al. (2012) [48]	Significant reduction in rates of admission in the youth-ACT sample. Percentage of adolescents with no admissions increased from 53% prior to referral to 83% post treatment	P-T^d^	Medical files	Reduction admissions:	1.0 (0.5, 1.6)
Godley et al. (2015) [52]	Significant fewer days spent in residential treatment, juvenile detention, and hospitals over the 12 month follow-up period compared to UCC	3, 6, 9, 12	Medical files	Reduction admission days:	0.3 (0.1, 0.6)
Schley et al. (2008) [42]	Comparison of psychiatric hospital admission rates and average number of days in the hospital prior to and after youth-ACT treatment showed that admission rates decreased with 17% at 3 month, 29% at 6 month, 28% at 9 month and 22% at 12 month follow-up	3, 6, 9, 12	Structured self-developed questionnaire	Reduction in hospital admissions days: 3 months: 6 months: 12 months:	1.6 (1.2, 2.1) 1.1 (0.7, 1.5) 0.7 (0.1, 1.2)

^a^Effect sizes were computed as Cohen’s d rounded to the first decimal place. Positive effect sizes represents improvement. Small (≥ 0.2–0.5); medium (> 0.5–0.8); large (> 0.8) [44]

^b^CI = Confidence interval

^c^NR = Not reported

^d^P-T = Pre-Post measurement was conducted

#### Frequency

Three studies examined the effect on frequency of admissions [42, 46, 48]. One study examined the frequency and duration of psychiatric hospital admissions during three-monthly intervals over a period of 12 months prior and post youth-ACT treatment [42]. This study showed that, with youth-ACT, the frequency of admissions decreased 7% at 3 month, 29.4% at 6 month, and 27.6% at 9 month follow-up. No significant effects were found at 12 months [42]. Another study found a decrease of admission rates (ES = 1.0) in patients who received youth-ACT [48]. A third study reported that youth-ACT resulted in a decrease in hospital admissions [46]. For this study ES could not be calculated because required data were not reported.

#### Duration

Table 4 shows that four studies examined the effect of youth-ACT on duration of hospital admissions [42, 46, 47, 52]. Reduction in duration of hospital admission was reported in all four studies. In one study small effect sizes were found for a decrease of days in hospitals at 12 month follow-up [52]. A second study found medium effect sizes for a decrease in duration of hospital admissions (ES = 0.5) and days spent in psychiatric institutions (ES = 0.6 [47]). Another study found large effect sizes at 3 month (ES = 1.6), 6 month (ES = 1.1) follow-up, and a medium effect size at 12 month (ES = 0.7) follow-up [42]. A fourth study reported that youth-ACT resulted in significantly shorter hospital admissions [46]. For this study ES could not be calculated because required data were not reported.

## Discussion

This review summarises the outcomes of 13 studies examining the effects of youth-ACT on severity of psychiatric symptoms, general functioning, and frequency and duration of psychiatric hospital admissions.

### Clinical implications

There are indications that youth-ACT is effective with respect to diminishing the severity of psychiatric symptoms in adolescents. Effect sizes range from small to large.

The single study that did not yield a significant effect was a RCT that found that youth-ACT had no additional effect if applied as a supplement to office-based mental healthcare [51]. This study consisted of an intervention that was limited to an average of only five to eight sessions. This low number of sessions may explain the lack of effect [39]. Because ACT in adults seems more effective in patients with severe problems [23], another explanation could be that the included patients in this study had relatively mild problems [39]. Also, it could be that ACT was compared to another intervention, in this particular case a behavioral therapeutic intervention, which was very effective with respect to substance abuse. In other words, there was no clear contrast between experimental and control group regarding therapeutic efforts.

#### Emotional problems

Some studies showed that youth-ACT is beneficial for adolescents with emotional problems [6, 8, 42, 43]. Studies concerning ACT in adults found effects on emotional problems that range from small (ES = 0.2 [20]) to medium (ES = 0.5 [28]). In youths, effects vary from small to large which could mean that ACT may be more effective in addressing emotional problems in children and adolescents.

Guidelines for emotional problems (anxiety or depression) in children and adolescents, for example the NICE guideline “Depression in children and young people” [62], do not provide recommendations with respect to youth-ACT. Children with emotional problems can be difficult to reach by outpatient care, because of avoidance (in case of anxiety) or depression (due to lack of energy, or loss of interest, for instance, in school, work or friends). Children with severe emotional problems have an increased risk of psychiatric hospitalization [63]. Youth-ACT teams can actively approach these children in their own living environment, instead of leaving them at home, without offering treatment, which may result in an increase in depression and anxiety, and ultimately, self-harm behaviors, increased parental stress, and hospitalization [64]. Youth-ACT might be a suitable approach for early screening, diagnosis, and treatment of care for children and adolescents with anxiety disorders or depression [64].

#### Behavioral problems

There is no evidence that youth-ACT is effective for disruptive and aggressive behaviors [6]. This conclusion is based on one study. If outreach treatment is needed, Multi Systemic Therapy (MST) [65, 66] or Multidimensional Family Treatment (MDFT) [67] may be more appropriate in accordance with the NICE guideline “Antisocial behavior and conduct disorders in children and young people” [68].

#### Psychotic problems

Two studies indicate that youth-ACT is effective in reducing psychotic symptoms [6, 40]. Effect sizes range from small to large. In adults, effects range from not significant (ES = 0.1 [20]) to medium (ES = 0.5 [22]). This may mean that in youth, ACT may be more effective. Children and adolescents with psychotic disorders have an increased risk of psychiatric hospitalization [69] and their long-term prognosis is often poorer than in adults [70]. Youth-ACT might play a key role in limiting long-term disability by providing early diagnostics and intervention [71]. The use of assertive case management for psychotic problems in adolescents is in accordance with existing guidelines, such as the NICE guideline “Psychosis and schizophrenia in children and young people” [14] and Orygen guideline “Australian clinical guidelines for early psychosis” [72].

#### Addiction problems

Youth-ACT appears effective in reducing cannabis use, and can be applied in case of care avoidance of children and adolescents [41, 49, 50, 52]. This conclusion is in accordance with the NICE guideline “Drug misuse in over 16s: psychosocial interventions” [73].

Unlike for adults, where effect sizes ranged from medium (ES = 0.5 [25]) to large (ES = 0.9 [25]; ES = 1.5 [24]), a majority of the studies in youths found no evidence that youth-ACT is effective for alcohol abuse. However, it has to be noted that only a limited number of studies examined these effects. Nevertheless, and similar as in adults [74], youth-ACT is used to support care-avoiding adolescents with severe alcohol abuse who do not benefit from other intensive treatment programs [3, 75]. The NICE guideline “Alcohol-use disorders: diagnosis, assessment and management of harmful drinking and alcohol dependence” recommends Assertive Outreach for adolescents [76]. Based on current evidence, the question arises whether it is appropriate to apply youth-ACT in adolescents with treatment resistant alcohol abuse. More research is needed.

Youth-ACT appears to improve general functioning in adolescents with severe psychiatric symptoms [6, 41–43, 46, 48, 52]. Effects seem comparable with studies investigating ACT in adults that found small (ES = 0.2 [25]; ES = 0.3 [21]), medium (ES = 0.6 [22]) and large (ES = 1.7 [24]) effects. Large significant effects on general functioning coincided with large effects on psychotic symptoms and mood disorders.

#### School attendance

There are indications that youth-ACT improves school attendance [6, 40, 41, 43, 46, 48]. It may be seen as encouraging that three studies with respect to school attendance found positive effects, since absenteeism is associated with an increase in severity of psychiatric symptoms, dropout from school, and unemployment [59, 77, 78].

#### Interpersonal relations

Youth-ACT may improve interpersonal relations [6]. Effects on family relations are small, however slightly larger, these effects are comparable with adults (ES = 0.3 [19]). Youth-ACT programs focus on participation in the community, since adolescents with severe psychiatric symptoms often have a small social network and weak social support, which can be attributed to a high levels of impairment in social functioning [36, 79].

#### Hospital admissions

Youth-ACT appears to reduce duration and frequency of psychiatric hospital admissions [42, 46–48, 52]. This is of interest, because children and adolescents with severe psychiatric symptoms are at a higher risk of being hospitalized than adults with similar problems [11]. Similar to adults where effects range from small (ES = 0.2 [23]), medium (ES = 0.4 [25]) to large (ES = 1.9 [27, 80]), youth-ACT may contribute to deinstitutionalization [1, 2] and higher cost-effectiveness. In addition, fewer hospital admissions may be associated with better social functioning, since adolescents are not “removed” from their social environment [71]. This is in line with the finding that youth-ACT may help to improve interpersonal relations [6].

### Strengths and limitations

This review has several strengths. First, it is the first review to date describing effectiveness of youth-ACT. Evidence has been summarized regarding current knowledge about its effects on psychiatric symptoms, general functioning, and hospital admissions. Second, studies were selected and assessed on their core elements of youth-ACT, to avoid missing relevant information.

Limitations pertain to the number and quality of studies published so far. However, despite this limitation, clear patterns are visible and unambiguous trends have been found in favor of youth-ACT.

According to the Oxford Centre of Evidence-Based Medicine grading system [38] all overall conclusions received a moderate strength of recommendation (grade B). Another drawback is that a majority of the studies were conducted in the United States which might hamper generalizability of findings to countries outside the United States [39, 81].

### Recommendations

Randomized controlled trials (RCTs) in different countries are needed to obtain grade A knowledge about the effect of youth-ACT. Such studies should also include children below the age of 12 years. The focus should be on a wide range of outcomes, including psychopathology and social functioning in several areas. Future studies should report on model fidelity to obtain a better insight into specific content of the youth-ACT program. The Dartmouth Assertive Treatment Scale (DACT) [5, 7] can be used for this purpose. Finally, although youth-ACT programs use a family approach, none of the studies provide detailed information about psychiatric and psychosocial problems of family members. Insight into these problems is needed, since such problems are likely to be present given familial aggregation of psychiatric disorders [82], and may influence treatment outcome.

## Conclusion

The findings of the studies included in this literature review are promising, despite the limitations described with respect to study designs. There are indications that youth-ACT is effective in reducing severity of psychiatric symptoms, improving general functioning, and reducing duration and frequency of psychiatric hospital admissions. Implementation of youth-ACT is high on the political and mental health agenda, which stresses the need for more research on its effectiveness using rigorous research designs.

## Additional file

Additional file 1: “Search strings review youth-ACT”. (DOCX 15 kb)

## Abbreviations

ACT: Assertive community treatment
AUS: Australia
CGAS: Children’s global assessment scale
CGI: Clinical global impression scale
CHE: Switzerland
DACTS: Dartmouth assertive treatment scale
DSM: Diagnostic systematic manual of mental disorders
EAHC: Executive agency for health and consumers
ES: Effect size
GAF: Global assessment of functioning
GAIN: Global appraisal of individual needs
GBR: Great Britain
HoNOSCA: Health of the nation outcome scales child and adolescents mental health
ISO: International Organization for Standardization
MDFT: Multidimensional family treatment
MST: Multi systemic therapy
NICE: The National Institute for health and care excellence
RCT: Randomized controlled trial
SCID-I: Structured clinical interview for DSM-IV axis I disorders
SD: Standard deviation
SIPS: Structured interview for prodromal syndromes
SOFAS: Social and occupational functioning assessment scale
TLFB: Timeline follow back
UCC: Usual continuing care
USA: United States of America
WHO: World Health Organization
