# Supplementary material for: The effect of youth assertive community treatment: a systematic PRISMA review
Source: BMC Psychiatry. 2017 Aug 2;17:284. doi: 10.1186/s12888-017-1446-4 (PMC5541424; doi:10.1186/s12888-017-1446-4)
Supplement: Additional file 1: — “Search strings review youth-ACT”. (DOCX 15 kb) [file 12888_2017_1446_MOESM1_ESM.docx]

**Search strings that were used in the review youth-ACT**

**Search string Cinahl**

| ("assertive community treatment" OR (MH "Community Mental Health Services+" AND (TI "act" OR AB "act" OR TI "assertive" OR AB "assertive"))) AND (MH "Child+" OR TI child* OR AB child* OR MH "Minors(legal)" OR TI minors OR AB minors OR MH "Puberty+" OR TI puberty OR AB puberty OR MH "Pediatrics+" OR TI paediatric* OR AB paediatric* OR TI pediatric* OR AB pediatric* OR MH "Adolescence+" OR TI adolescen* OR AB adolescen* OR TI preschool* OR AB preschool* OR TI teenager* OR AB teenager* OR TI teen* OR AB teen* OR TI youth* OR AB youth* OR TI girlhood OR AB girlhood OR TI girl OR AB girl OR TI girls OR AB girls OR TI boyhood OR AB boyhood OR TI boy OR AB boy OR TI boys OR AB boys OR TI “school age” OR AB “school age” OR TI “school-aged” OR AB “school-aged” OR TI schoolchild* OR AB schoolchild* OR TI kid OR AB Kid OR TI kids OR AB kids OR TI underage* OR AB underage* OR TI Juvenile* OR AB Juvenile*) |
| --- |

**Search string PsychInfo**

Choose **advanced search** **in Psychinfo** and build the searchbuilder.

| **Row 1: (#1) =**  (child* or Minors or Puberty or Pediatrics or Paediatrics or Adolescen* or Preschool* or Teenager or Teenager* or Teen* r Youth* or Girlhood or Girl* or Girl or boyhood or boy or Boy* or school age or schoolchild* or kid or kids or underage* or Juvenile*).ab,ti.  **Row 2: (#2) =** (ACT or assertive).ab,ti. and (community mental health/ r exp community mental health services/)  **Row 3: (#3) =** Assertive community treatment.ab,ti.  **Row 4 (#4) =** 2 OR 3  **Row 5 (#5) =** 1 AND 4 |
| --- |

**Search string The Cochrane Library**

Step 1: Choose advanced search in Cochrane database

Step 2: Choose Medical Terms (MeSH) om MeSH termen in te voeren. Press Lookup to

go to the next field.

Step 3: Go to screen “ADD to search manager”

Step 4: MeSH term is now added to the searchbuilder.

| **Row 1: (#1) =** Child  **Row 2: (#2) =** Community Mental Health Services  **Row 3: (#3) =** Adolescent  **Row 4 (#4) =** Minors  **Row 5 (#5) =** Puberty  **Row 6 (#6) =** Pediatrics  **Row 7 (#7) =** assertive community treatment:ti,ab,kw  **Row 8 (#8) =** ACT:ti,ab,kw OR Assertive:ti,ab,k  **Row 9 (#9) =**  (child* or minors or puberty or Paediatric* or Pediatric* or Adolescent* or Preschool* or Teenager or Teenagers or Teen or Teens or Youth* or Girlhood or Girl or Girls or Girl or Boyhood or Boy or Boys or school age or School-aged or Schoolchild* or Kid or Kids or Underage* or Juvinile*):ti,ab,kw  **Row 10 (#10) =** #2 AND #8  **Row 11 (#11) =** #1 OR #3 OR #4 OR #5 OR #6 OR #9  **Row 12 (#12)** = #9 or #11  **Row 13 (#13) =** (#7 or #10) and #12  **Please note: row 1 to 8 are mesh terms.** |
| --- |

**Search string Pubmed**

| ("assertive community treatment"[tiab]OR ("Community Mental Health Services"[Mesh] AND (act[tiab] OR assertive[tiab]))) AND ("Child"[Mesh] OR child*[tiab] OR "Minors"[Mesh] OR “minors”[tiab] OR "Puberty"[Mesh] OR "puberty"[tiab] OR "Pediatrics"[Mesh] OR paediatric*[tiab] OR pediatric*[tiab] OR "Adolescent"[Mesh] OR adolescen*[tiab] OR preschool*[tiab] OR "teenager"[tiab] OR "teenagers"[tiab] OR "teen"[tiab] OR "teens"[tiab] OR youth*[tiab] OR "girlhood"[tiab] OR "girl"[tiab] OR "girls"[tiab] OR "boyhood"[tiab] OR "boy"[tiab] OR "boys"[tiab] OR "school age"[tiab] OR "school-aged"[tiab] OR schoolchild*[tiab] OR "kid"[tiab] OR "kids"[tiab] OR underage*[tiab] OR juvenile*[tiab]) |
| --- |
